# The impact of geriatric nutritional risk index on one-year outcomes in hospitalized elderly patients with heart failure

**DOI:** 10.3389/fcvm.2023.1190548

**Published:** 2023-05-30

**Authors:** Masakazu Miura, Shinichi Okuda, Kazuhiro Murata, Yutaka Ohno, Satoshi Katou, Fumiaki Nakao, Takeshi Ueyama, Takeshi Yamamoto, Yasuhiro Ikeda

**Affiliations:** ^1^ Department of Rehabilitation, Yamaguchi Prefectural Grand Medical Center, Hofu, Japan; ^2^ Division of Nursing and Laboratory Science, Yamaguchi University Graduate School of Medicine, Ube, Japan; ^3^ Department of Cardiology, Yamaguchi Prefectural Grand Medical Center, Hofu, Japan

**Keywords:** heart failure, geriatric nutritional risk index (GNRI), short performance physical battery, barthel index (BI), cardiac rehabilitation (CR)

## Abstract

**Background:**

Strategies that accurately predict outcomes in elderly heart failure (HF) patients have not been sufficiently established. In previous reports, nutritional status, ability to perform activities of daily living (ADL), and lower limb muscle strength are known prognostic factors associated with cardiac rehabilitation (CR). In the present study, we investigated which CR factors can accurately predict one-year outcomes in elderly patients with HF among the above factors.

**Methods:**

Hospitalized patients with HF over 65 years of age from January 2016 to January 2022 were retrospectively enrolled in the Yamaguchi Prefectural Grand Medical (YPGM) Center. They were consequently recruited to this single-center retrospective cohort study. Nutritional status, ADL, and lower limb muscle strength were assessed by geriatric nutritional risk index (GNRI), Barthel index (BI), and short physical performance battery (SPPB) at discharge, respectively. One year after discharge, the primary and secondary outcomes were evaluated by all-cause death or HF readmission and major adverse cardiac and cerebrovascular events (MACCE), respectively.

**Results:**

Overall, 1,078 HF patients were admitted to YPGM Center. Of those, 839 (median age 84.0, 52% female) met the study criteria. During the follow-up of 228.0 days, 72 patients reached all-cause death (8%), 215 experienced HF readmission (23%), and 267 reached MACCE (30%: 25 HF death, six cardiac death, and 13 strokes). A multivariate Cox proportional hazard regression analysis revealed that the GNRI predicted the primary outcome (Hazard ratio [HR]: 0.957; 95% confidence interval [CI]: 0.934–0.980; *p* < 0.001) and the secondary outcome (HR: 0.963; 95%CI: 0.940–0.986; *p* = 0.002). Furthermore, a multiple logistic regression model using the GNRI most accurately predicted the primary and secondary outcomes compared to those with the SPPB or BI models.

**Conclusion:**

A nutrition status model using GNRI provided a better predictive value than ADL ability or lower limb muscle strength. It should be recognized that HF patients with a low GNRI at discharge may have a poor prognosis at one year.

## Introduction

Heart failure (HF) is a leading cause of morbidity and mortality, with a prevalence of 23 million worldwide (1–3). The number of patients is increasing in our super-aging society (4). Although new pharmacological (5, 6) and non-pharmacological therapies (7, 8) have been developed in the past decade, prognostic improvement in HF patients remains inadequate (9). Several studies have reported that elderly HF patients frequently present malnutrition (10), frailty (10, 11), sarcopenia (12, 13), and malnutrition resulting in poor prognosis (14, 15). In this regard, non-invasive interventions, including nutritional assessment and cardiac rehabilitation (CR), have recently attracted attention.

The Geriatric Nutritional Risk Index (GNRI), consisting of body mass index (BMI) and albumin, is a simple and versatile nutritional assessment tool in HF patients associated with all-cause or cardiac death (16–19). Previous reports have shown that a lower GNRI of less than 92 was associated with poor mortality (20, 21). On the other hand, a short physical performance battery (SPPB) provides a reasonable lower limb muscle-strength assessment value (22) and is also associated with physical balance ability. The SPPB consists of a 12-point scale, with even a one-point reduction representing a significant decline in lower limb physical function (23). The lower value of SPPB below seven is diagnosed as frailty and is associated with frequent rehospitalization and poor mortality (24–26). Moreover, the estimated activity of daily living (ADL) has been shown as another prognostic factor in HF patients, i.e., the Barthel index (BI) (27), which consists of 100 point scale assessing eating, grooming, ability to walk, and climb stairs, and management of toileting. HF patients with a low BI of less than 85 have been reported to go a poor prognosis (28).

In recent years, GNRI, BI, and SPPB have been used as rehabilitation indicators in CR to represent nutritional indices, ADL assessment, and lower limb physical activity capacity, respectively. Of these three assessment measures used in CR, it has not yet been established which is the most predictive outcome in elderly patients with HF (29–32).

This study examined which multivariate models using GNRI, SPPB, and BI best predicted outcomes at one year in hospitalized HF patients.

## Method

### Study population

From January 1, 2016, to January 31, 2022, at the Yamaguchi Prefectural Grand Medical (YPGM) Center, patients over 65 years, who were admitted to the emergency room due to acute decompensated HF (ADHF), hospitalized for treatment and underwent CR, were enrolled in this retrospective cohort study. The diagnosis of HF was made according to the Heart Failure Guidelines of the American Heart Association (AHA)/American College of Cardiology (ACC) (32) and the European Society of Cardiology (ESC) (31). Exclusion criteria were defined as follows: (1) in-hospital death, (2) lack of physical functional evaluation, (3) lost follow-up, and (4) no echocardiographic assessment. As part of comprehensive CR, we provided exercise therapy and nutritional guidance to all enrolled HF patients during hospitalization. Outpatient rehabilitation is provided to less than 5% of the enrolled HF inpatients. The study was performed per the Declaration of Helsinki and approved by the local institutional board at the YPGM Center (ID: 2022-013J). We applied the opt-out form to obtain informed consent by posting the document on our hospital website. Patients who did not want to participate in this study were instructed to contact the director.

### Assessment with GNRI, SPPB, and BI

We evaluated the GNRI calculated as (14.89 × albumin) + 41.7 × [body weight(kg)/ideal body weight(kg)] (20) just before discharge. SPPB assessing a lower-limb function consists of gait speed (4-meter walk time), 5-second chair standing time, and balance (closing the legs, semi-tandem, and tandem). Each test scored from 0 to 4, with a total score ranging from 0 to 12 points; a higher score indicates a better lower limb function (22). BI measured the capacity in ADL, the BI is a major index with ten items, and the total BI score ranged between 0 and 100 points, with higher values indicating a higher level of independent physical state (27). The cut-off value of BI was identified that 85 points, 60 points, and 40 points, respectively (28). SPPB and BI were evaluated within five days of discharge, and body weight and serum albumin for calculating GNRI were assessed just before discharge.

### Echocardiographic study

A comprehensive echocardiographic examination was performed with the patient in stable condition within two weeks of admission. Two-dimensional measurements, including left ventricular end-diastolic dimension (LVDd), left ventricular end-systolic dimension (LVDs), and left atrial dimension (LAD), were obtained according to the recommendations of the American Society of Echocardiography (33). Apical four- and two-chamber views were used for calculating LVEF using biplane disk methods. The index, e′, was measured using tissue Doppler imaging, and the ratio of E-wave to e′ (E/e′) was calculated using the mean of the septal and lateral velocities (33). Trans-tricuspid pressure gradient (TR-PG) was measured using continuous-wave Doppler echocardiography. Right atrial pressure was estimated from the inferior vena cava (IVC) diameter and collapsibility. The estimated pulmonary arterial pressure (ePAP) was calculated as the sum of TR-PG and right atrial pressure.

### Baseline clinical characteristics, medication, and physical functional assessment

The baseline clinical characteristics were obtained from the electrical medical records. These characteristics include the following parameters; age, sex, living alone, returning home, nursing care insurance, history of HF, etiology of HF, and New York Heart Association (NYHA) functional classification on admission and at the time of discharge. The etiology of HF patients was classified from the medical record into seven categories: ischemic heart disease (IHD), valvular heart disease (VHD), cardiomyopathy (CM), hypertensive heart disease (HHD), arrhythmia (atrial fibrillation [Afib] and complete atrioventricular block [CAVB]), other [chronic kidney disease (CKD) and anemia], and uncertain. HF co-morbidities were evaluated for the presence of hypertension, diabetes mellitus, AFib, CKD (defined as eGFR <60 ml/min/1.73 m^2^), chronic obstructive pulmonary disease (COPD), stroke, and orthopedic disease. Usage of the following standard medications for HF was reviewed at discharge: angiotensin-converting enzyme inhibitors (ACE-I)/angiotensin II receptor blockers (ARB)/angiotensin receptor neprilysin inhibitors (ARNI), *β*-blockers, tolvaptan, loop diuretics, mineralocorticoid receptor antagonists (MRAs), and sodium-glucose cotransporter 2 (SGLT2) inhibitors. The laboratory data were also obtained from medical records, including B-type natriuretic peptide (BNP) at admission, albumin, hemoglobin, creatinine, and estimated glomerular filtration rate (eGFR). Physical functions were evaluated five days before discharge. These include the SPPB test, BI, a handgrip test by a grip strength meter (T.K.K.5401 GRIP-D; Takei, Tokyo, Japan), and the quadriceps isometric strength (QIS) test by a handheld dynamometer (MT-100 mobile; Sakai Med, Tokyo, Japan) (34). Exercise tolerance was evaluated by the 6 min walking test (6MWT) (35).

### Clinical outcomes and follow-up period

The primary outcome was evaluated as a composite of all-cause death or HF readmission one year after discharge. The secondary outcome was assessed as major adverse cardiac and cerebrovascular events (MACCE), i.e., cardiac death, HF death, HF readmission, acute myocardial infarction, unstable angina, aortic dissection, and stroke at one year. Medical information regarding one-year outcomes was collected from electric charts and by letter when available.

### Statistical analysis

Statistical analysis was performed using the EZR on R commander (version 1.37) (36). Categorical variables were expressed as numbers, and percentage (%) or continuous variables were expressed as means ± standard deviation or median [interquartile range (IQR): 25th to 75th percentiles]. We calculated the correlation coefficient between GNRI, SPPB, and BI using Pearson's or Spearman's rank correlation coefficient.

The univariate and multivariate Cox proportional hazard regression analyses identified predictors of primary and secondary outcomes. Independent variables for multiple modeling were selected from predictive factors with *p* < 0.10 using the univariate analysis and previously reported predictive factors, i.e., age, sex, LVEF, SPPB, BI, GNRI, and the natural logarithm of BNP. Since the variance of BNP in HF patients is extremely large and does not represent a normal distribution, its natural logarithm (Log BNP) was used for multivariate analysis. A stepwise variable reduction method was then used for the multivariate modeling. Results were provided as hazard ratio (HR), 95% confidence interval (CI), and *p*-value. When the multivariate analysis identified the predictors of continuous variables, a receiver operating characteristic (ROC) analysis was employed to determine the optimal cut-off value acting as independent predictive factors, followed by the sensitivity, the specificity, and the area under the curve (AUC). Event-free ratios were estimated by the Kaplan– Meier method and compared by the log-rank test.

Moreover, we considered three multivariate prognostic risk score models. The AUC was described to compare the prognosis index of GNRI, SPPB, and BI for predicting all-cause death or HF readmission and MACCE. Three prognosis risk score models adjusted for age, sex, and LVEF were developed to compare GNRI, SPPB, and BI based on the regression coefficient (37).

A *p*-value of less than 0.05 indicated a statistically significant.

## Results

### Patient clinical characteristics

**Figure 1:**
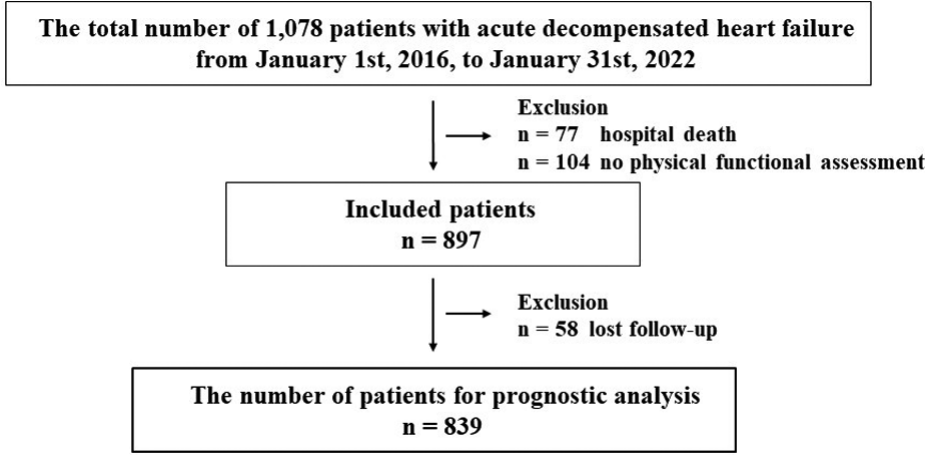
Flowchart of this study in patients with acute decompensated heart failure.

Overall, 1,078 patients with ADHF were admitted to the YPGM Center, as shown in Figure 1. Of those, 181 met the exclusion criteria. Briefly, 77 died in hospital treatment, 104 were not evaluated for physical functional assessment, and 58 patients were lost to follow-up. Ultimately, 839 patients were analyzed. The median age of included patients was 84.0 (IQR: 78.0–89.0), the prevalence of females was 436 (52%), and the length of hospital stay was 20.0 (IQR: 14.0–28.0) days in Table 1. We diagnosed 233 (28%) patients with HF with reduced LVEF (LVEF < 40%: HFrEF), 153 (18%) patients with HF with mildly reduced LVEF (LVEF 40%–49%: HFmrEF), and 453 (54%) patients with HF with preserved LVEF (LVEF ≥ 50%: HFpEF) (31, 32).

**Table 1:** Baseline clinical characteristics.

	Overall
	(*n* = 839)
Age, years	84.0 (78.0–89.0)
Female	436 (52)
BMI, kg/m^2^	20.2 (18.0–22.8)
Living alone	194 (23)
Returning home	593 (71)
Nursing care insurance	364 (43)
History of HF	323 (39)
NYHA II/III/IV (on admission)	80/223/536 (10/27/63)
NYHA I/II/III/IV (at discharge)	551/245/32/11 (66/29/4/1)
Hospital stay, days	20.0 (14.0–28.0)
Etiology	
IHD	177 (21)
VHD	258 (31)
CM	97 (12)
HHD	85 (10)
Arrhythmia (AFib and CAVB)	121 (14)
Other (CKD, anemia)	107 (13)
Uncertain	24 (3)
Co-morbidities	
Hypertension	578 (69)
Diabetes mellitus	292 (35)
AFib	333 (40)
CKD (eGFR < 60 ml/min/1.73 m^2^)	659 (79)
COPD	135 (16)
Stroke	150 (18)
Orthopedic disease	245 (29)
Medication	
ACE-I/ARB/ARNI	531 (63)
*β*-blocker	556 (66)
Tolvaptan	316 (38)
Loop diuretics	503 (60)
MRAs	402 (48)
SGLT2 inhibitors	115 (14)
Echocardiography	
LVEF, %	51.0 (37.4–63.0)
HFrEF	223 (28)
HFmrEF	153 (18)
HFpEF	453 (54)
LVDd, mm	50.0 (44.0–56.0)
LVDs, mm	35.0 (29.0–45.0)
LAD, mm	43.0 (37.0–48.0)
E/e′	15.8 (11.9–21.7)
TR-PG, mmHg	27.0 (21.5–34.1)
ePAP, mmHg	32.0 (27.0–40.0)
IVC, mm	14.0 (11.0–17.0)
Laboratory data	
BNP, pg/ml	542.0 (304.3–964.9)
Log BNP	2.73 (2.48–2.98)
Albumin, g/dl	3.3 (3.0–3.6)
Hemoglobin, g/dl	11.2 (9.9–12.6)
Creatinine, mg/dl	1.10 (0.83–1.49)
eGFR, ml/min/1.73 m^2^	43.0 (30.0–56.5)
GNRI	87.8 (80.8–95.1)
Physical function	
SPPB, points	7 (4–10)
QIS, Nm/kg	0.62 (0.46–0.78)
Handgrip, kg	14.0 (9.2–19.5)
BI, points	80.0 (60.0–90.0)
6MWT	240.0 (109.5–320.0)

Values were shown as median [interquartile range (IQR): 25th to 75th percentiles] and *n* (%). BNP is admission data, and the medication is prescription data at discharge. BMI, body mass index; HF, heart failure; NYHA, New York Heart Association; IHD, ischemic heart disease; VHD, valvular heart disease; CM, cardiomyopathy; HHD, hypertensive heart disease; AFib, atrial fibrillation; CAVB, complete atrioventricular block; CKD, chronic kidney disease; COPD, chronic obstructive pulmonary disease; ACE-I/ARB/ARNI, angiotensin-converting enzyme inhibitor/angiotensin II receptor blocker/angiotensin receptor neprilysin inhibitor; MRAs, mineralocorticoid receptor antagonists; SGLT2, sodium glucose cotransporter 2; LVEF, left ventricular ejection fraction; HFrEF, heart failure with reduced ejection fraction; HFmrEF, heart failure with mildly reduced ejection fraction; HFpEF, heart failure preserved ejection fraction; LVDd, left ventricular diameter end-diastolic diameter; LVDs, left ventricular end-systolic diameter; LAD, left atrial dimension; TR-PG, transtricuspid-pressure gradient; ePAP, estimated pulmonary arterial pressure; IVC, inferior vena cava; BNP, B-type natriuretic peptide; eGFR, estimated glomerular filtration rate; GNRI, geriatric nutritional risk index; SPPB, short physical performance battery; QIS, quadriceps isometric strength; BI, barthel index; 6MWT, 6 min walking distance.

### One-year outcomes of the analyzed ADHF patients

The median follow-up was 228.0 days. During the follow-up period, 72 patients reached all-cause death (8%), 215 experienced HF readmission (24%), and 267 reached MACCE (30%: 25 HF death, six cardiac death, and 13 strokes).

Univariate analysis of the primary outcome, i.e., all-cause death or HF readmission, selected 22 variables with a *p*-value of less than 0.10, including age, sex, LVEF, Log BNP, GNRI, SPPB, and BI. Multivariate Cox proportional hazard regression analysis revealed that the independent predictors were the history of HF, nursing care insurance, arrhythmia (etiology), LAD, Log BNP, GNRI, and BI. SPPB was not, as shown in Table 2.

**Table 2:** Univariate and multivariate cox proportional hazard analyses to predict All-cause death or HF readmission after discharge of 839 patients with ADHF.

Variables	Univariate		Multivariate	
	HR(95%CI)	*P* value	HR(95%CI)	*P* value
Age	1.027 (1.011–1.043)	<0.001		
Male sex	0.988 (0.786–1.241)	0.917		
History of HF	2.727 (2.164–3.436)	<0.001	2.019 (1.351–3.019)	0.001
NYHA I (at discharge)	reference			
NYHA II	1.546 (1.211–1.973)	<0.001		
NYHA III	1.987 (1.189–3.318)	0.009		
NYHA IV	4.564 (2.138–9.745)	<0.001		
Nursing care insurance	1.228 (0.977–1.542)	0.078	0.521 (0.329–0.825)	0.005
Hypertension (etiology)	0.628 (0.399–0.988)	0.044		
Arrhythmia (etiology)	0.540 (0.368–0.793)	0.002	0.464 (0.224–0.961)	0.039
VHD (etiology)	1.281 (1.009–1.628)	0.042		
SGLT2 inhibitor	0.654 (0.451–0.948)	0.025		
Tolvaptan	1.345 (1.069–1.692)	0.011		
Loop diuretic	1.694 (1.321–2.172)	<0.001		
E/e′	1.014 (1.004–1.026)	0.009		
ePAP	1.027 (1.017–1.037)	<0.001		
IVC	1.033 (1.009–1.057)	0.006		
LAD	1.017 (1.003–1.030)	0.014	1.036 (1.010–1.062)	0.006
LVDd	1.022 (1.008–1.036)	0.002		
LVDs	1.021 (1.009–1.033)	<0.001		
LVEF	0.985 (0.978–0.992)	<0.001		
TR-PG	1.023 (1.013–1.033)	<0.001		
Log BNP	2.805 (2.064–3.813)	<0.001	2.068 (1.154–3.706)	0.015
Creatinine	1.062 (1.003–1.124)	0.039		
eGFR	0.987 (0.983–0.995)	<0.001		
GNRI	0.962 (0.952–0.973)	<0.001	0.957 (0.934–0.980)	<0.001
Hemoglobin	0.856 (0.805–0.911)	<0.001		
SPPB	0.939 (0.909–0.970)	<0.001		
BI	0.989 (0.984–0.993)	<0.001	0.981 (0.971–0.991)	<0.001
QIS	0.661 (0.409–1.069)	0.091		
Grip strength	0.965 (0.948–0.982)	<0.001		
6MWT	0.998(0.996–0.999)	<0.001		

Table 2 shows the results of the univariate and multivariate Cox proportional hazard analyses. The *p*-value less than 0.10, age, sex, LVEF, SPPB, BI, GNRI, and Log BNP were listed in this table. A GNRI consists of BMI and albumin; therefore, these variables were excluded from multiple modeling. HF, heart failure; ADHF, acute decompensated heart failure; HR, hazard ratio; CI, confidence interval. The other abbreviations are the same as listed in Table 1.

Second, for analysis of secondary outcome, i.e., MACCE, univariate analysis revealed 19 variables with a *p*-value of less than 0.10, including age, sex, LVEF, Log BNP, GNRI, SPPB, and BI. Multivariate Cox proportional hazard regression analysis revealed the NYHA (at discharge), VHD (etiology), LAD, LVEF, Log BNP, eGFR, and GNRI as independent predictors, while SPPB and BI were not, as shown in Table 3.

**Table 3:** Univariate and multivariate cox proportional hazard analyzes to predict MACCE after discharge of 839 patients with ADHF.

Variables	Univariate		Multivariate	
	HR(95%CI)	*P* value	HR(95%CI)	*P* value
Age	1.019 (1.003–1.036)	0.019		
Male sex	0.983 (0.773–1.250)	0.890		
History of HF	2.865 (2.244–3.658)	<0.001		
NYHA I (at discharge)	reference			
NYHA II	1.360 (1.048–1.764)	0.020	1.590 (1.035–2.442)	0.034
NYHA III	1.832 (1.061–3.264)	0.030	1.195 (0.363–3.938)	0.770
NYHA IV	3.484 (1.427–8.506)	0.006	23.91 (6.330–90.29)	<0.001
Hypertension (etiology)	0.587 (0.359–0.959)	0.033		
Arrhythmia (etiology)	0.580 (0.392–0.858)	0.006		
VHD (etiology)	1.351 (1.052–1.735)	0.018	1.526 (1.010–2.305)	0.045
Tolvaptan	1.343 (1.055–1.711)	0.017		
Loop diuretic	1.708 (1.314–2.219)	<0.001		
E/e′	1.014 (1.003–1.026)	0.015		
ePAP	1.027 (1.017–1.038)	<0.001		
IVC	1.035 (1.011–1.061)	0.005		
LAD	1.024 (1.010–1.038)	<0.001	1.051 (1.022–1.080)	<0.001
LVDd	1.026 (1.011–1.041)	<0.001		
LVDs	1.024 (1.011–1.036)	<0.001		
LVEF	0.985 (0.977–0.992)	<0.001	0.984 (0.970–0.998)	0.024
TR-PG	1.024 (1.013–1.034)	<0.001		
Log BNP	2.896 (2.095–4.003)	<0.001	1.954 (1.035–3.688)	0.039
Creatinine	1.055 (0.990–1.124)	0.098		
eGFR	0.991 (0.984–0.997)	0.003	0.986 (0.974–0.997)	0.017
GNRI	0.971 (0.960–0.982)	<0.001	0.963 (0.940–0.986)	0.002
Hemoglobin	0.902 (0.846–0.962)	0.002		
SPPB	0.973 (0.940–1.007)	0.114		
BI	0.994 (0.989–0.998)	0.008		
Grip strength	0.975 (0.956–0.993)	0.006		
6MWT	0.998 (0.997–0.999)	0.004		

Table 3 shows the results of the univariate and multivariate Cox proportional hazard analyses. The *p*-value less than 0.10, age, sex, LVEF, SPPB, BI, GNRI, and Log BNP were listed in this table. A GNRI consists of BMI and albumin; therefore, these variables were excluded from multiple modeling. MACCE, major adverse cardiac and cardiovascular events. The other abbreviations are the same as listed in Tables 1, 2.

**Figure 2:**
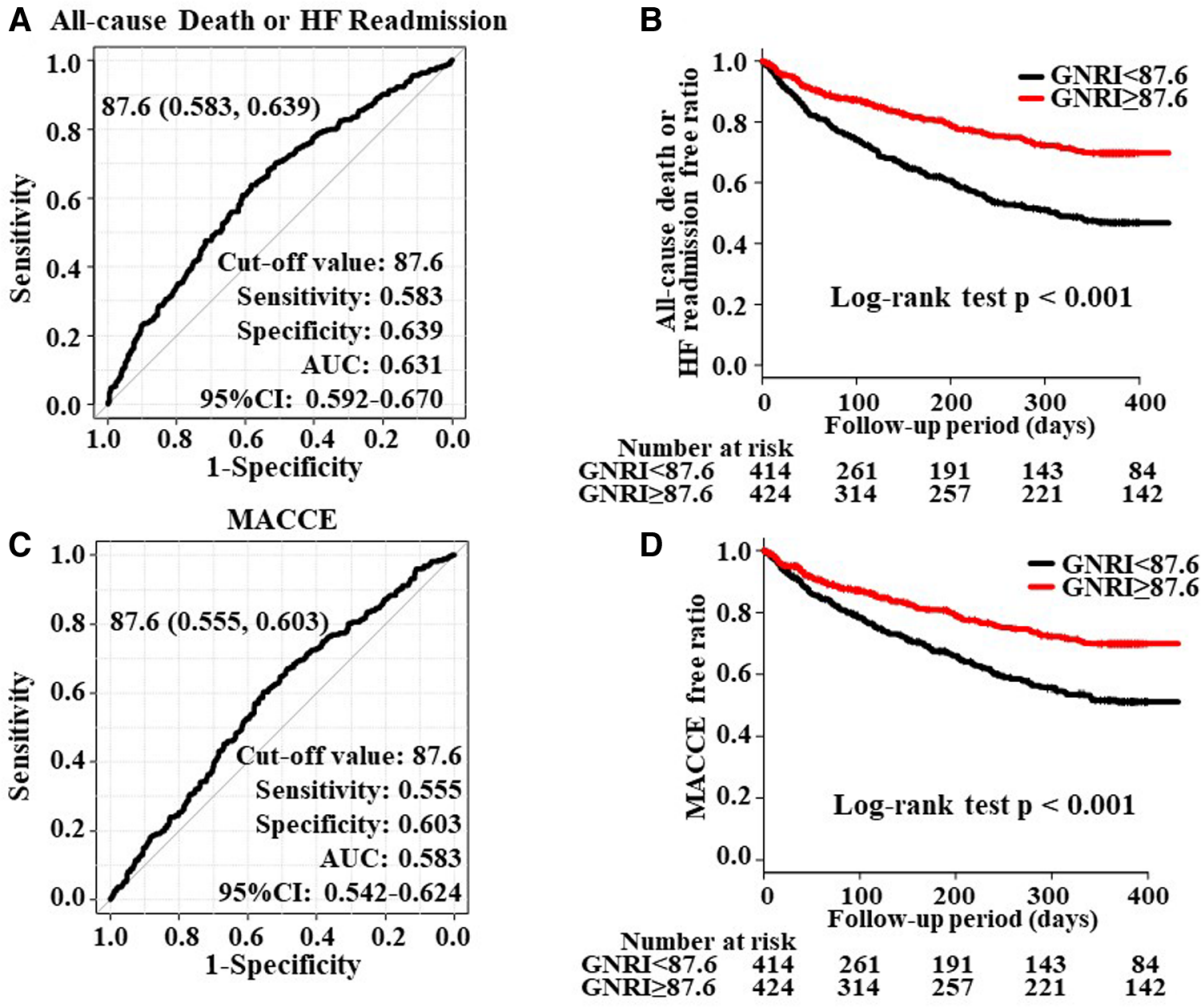
Panels (**A, C**) show the ROC curve to predict the cut-off value of all-cause death, HF readmission, and MACCE overall HF patients (*n* = 839). Panels (**B, D**) show the Kaplan-Meier curve to evaluate the cumulative incidences of all-cause death or HF readmission and MACCE.

The ROC analysis of the primary outcome revealed the maximum AUC as 0.631 (95% CI; 0.592–0.670) when the cut-off value of GNRI was set to 87.6 (Figure 2A). The Kaplan–Meier curve showed a significantly higher incidence of the primary endpoint in patients with GNRI < 87.6 than in those with GNRI ≥ 87.6 (Figure 2B).

The ROC analysis of the secondary endpoint revealed the maximum AUC as 0.583 (95% CI; 0.542–0.624; Figure 2C) and a similar Kaplan-Meier curve to the primary endpoint (Figure 2D).

### Comparison with multivariate predicting risk score models using GNRI, SPPB, and BI

**Figure 3:**
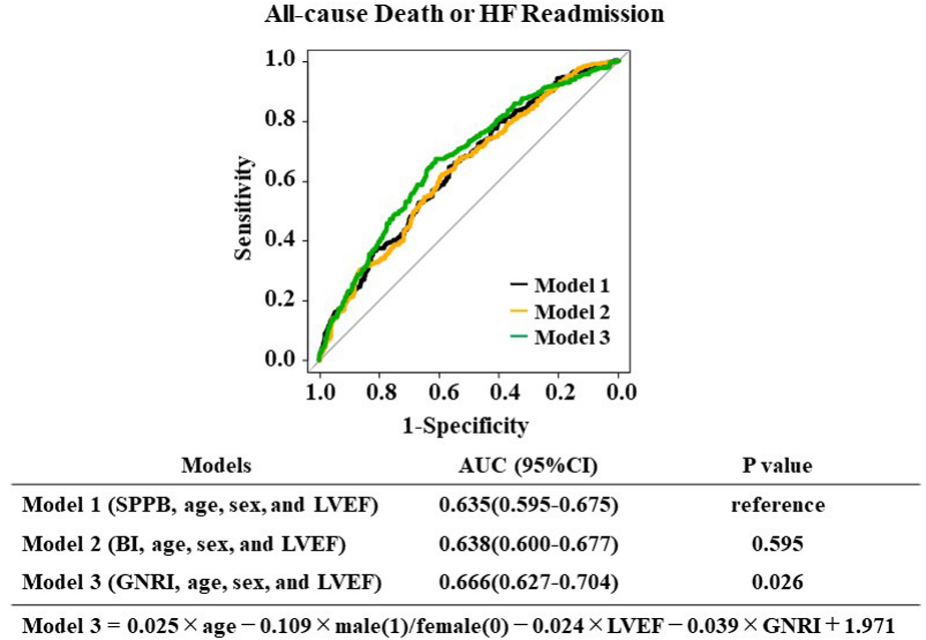
The risk score models for predicting all-cause death or HF readmission and MACCE were compared using the area under the receiver operating characteristic curves.

We then created multiple logistic models using GNRI, SPPB, and BI with age, sex, and LVEF and examined which model could provide the largest AUC. As shown in Figure 3, the GNRI model (Model 3) presented the largest AUC compared to those of the SPPB (Model 1) and the BI (Model 2) for predicting the primary endpoint. On the other hand, analysis for predicting the secondary endpoint did not reach statistical significance among the three groups (Model 4:SPPB, Model 5:BI, Model 6:GNRI), as presented in Supplementary Figure S1.

### Relationships between GNRI, SPPB, BI, and one-year outcomes

**Figure 4:**
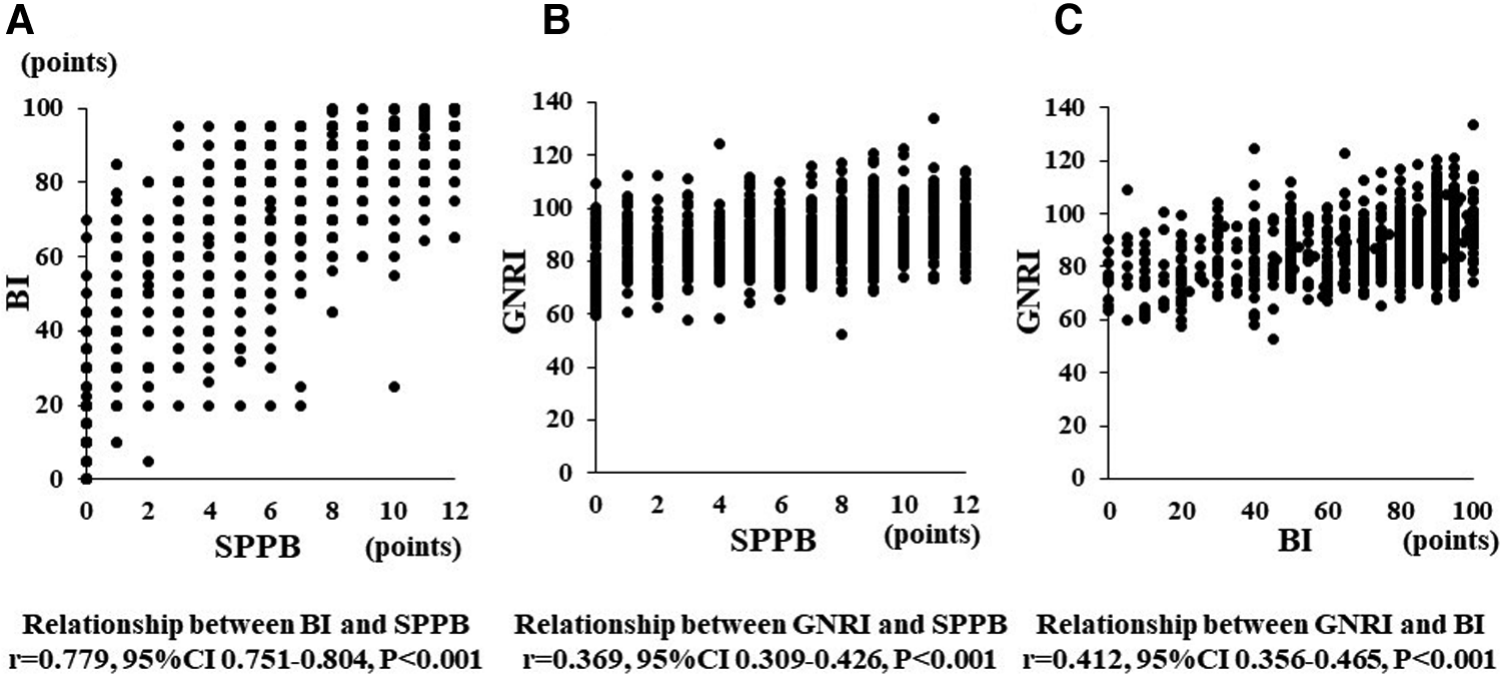
Panel (**A–C**) show a relationship between BI and SPPB, GNRI and SPPB, and GNRI and BI, respectively.

Malnutrition, frailty, and low ADL may coexist in patients with poor prognoses of HF. Although SPPB, BI, and GNRI, which were analyzed in this study, each represent these indices, we thought it necessary to investigate their relationship. Therefore, we examined the correlation among GNRI, SPPB, and BI as continuous variables (Figure 4). SPPB and BI showed a high correlation coefficient (*r* = 0.779) compared to that between GNRI and SPPB (*r* = 0.369) or GNRI and BI (*r* = 0.412). These data suggest that GNRI is highly independent of other CR-associated factors.

## Discussion

In the present study, we investigated whether any of the three poor prognostic factors in elderly HF patients, including low nutrition, frailty, and decline in ADL, could most accurately predict prognosis. We found that the GNRI, a simple and versatile measure of nutrition, was the most accurate and strongly correlated with prognosis one year after discharge. To the best of our knowledge, the finding has not been reported before, and we believe it is extremely important for predicting the prognosis of elderly HF patients.

### GNRI, a good measure of the nutritional index in hospitalized elderly HF patients

Several indicators of malnutrition in elderly HF patients have been reported. These include albumin (16), BMI (38), MNA-SF (39), and the Global Leadership Initiative on Malnutrition (GLIM) criteria (40). Nutritional assessment of HF patients has been performed using these indices, and it has been reported that malnutrition is strongly associated with adverse outcomes (14, 15). On the other hand, which of the nutritional indices is best is controversial and was not investigated in this study. For example, the MNA-SF is a tool to evaluate nutritional status over the past three months (39). Although MNA-SF is considered a good indicator for outpatient (41), it is not suitable for accurate prediction during acute exacerbations of HF since body weight can change quickly due to systemic edema and congestion. In this regard, we expected that GNRI would be a good predictor in hospitalized patients, based on previous reports in hospitalized HFpEF patients and our previous report on its usefulness as a prognostic indicator in VHD patients (21, 42). We found that the GNRI predicted all-cause mortality or HF hospitalization one year after discharge with high predictive accuracy. Although the cut-off value of the GNRI for predicting outcomes was even lower than in previous reports, it possibly related to the high proportion of very elderly patients in this study.

### Unique correlations between GNRI and other CR-associated factors in elderly HF patients

In the ROC analysis conducted in this study, a multivariate model was created by adjusting GNRI, SPPB, and BI, indicators used in CR, for age, sex, and LVEF. This method makes more systematic predictions by adding variables expected to be affected by the ROC analysis, usually performed univariately as multivariate factors (21). As a result, we found that the model using GNRI was the most accurate in predicting prognosis. In a previous study (21), GNRI was reported to be a good prognostic indicator in patients with HFpEF. The present study confirmed the benefit of the GNRI even when LVEF values are included in the prediction model, suggesting that the GNRI is an accurate predictor for patients with HFpEF and other categories of elderly HF patients. However, there is room for further investigation as to why the model using GNRI obtained the highest AUC.

The fact that GNRI, measured once before discharge, accurately predicts patient prognosis one year later is very interesting. When we examined the correlation between GNRI, SPPB, and BI in elderly HF patients, we found a strong correlation between SPPB and BI. On the other hand, the correlation coefficients between GNRI and SPPB, or GNRI and BI, were not high enough compared to that between SPPB and BI. These findings suggest that GNRI does not simply reflect ADL ability or lower muscle strength but is associated with whole-body nutritional status. Very few reports showed that CR improved GNRI value (26, 43). In contrast, SPPB and BI have been clearly shown to improve with the continuous effort of CR (44). In this sense, HF patients with high BMI are known to have a relatively preserved prognosis, i.e., an obesity paradox (14). It is suggested that GNRI is not a simple nutritional assessment tool but also a general prognostic indicator of HF patients.

In contrast, the secondary outcome, MACCE, did not differ among the three risk score models. Previous studies have suggested that non-cardiovascular death and non-cardiovascular events are associated with outcomes in elderly HF patients (45, 46). This study's high median patient age (84 years) may have contributed to the analysis results.

Recently, as a new pharmacologic therapy for chronic HF, several large clinical trials have held great promise for preventing HF hospitalization in patients with HFpEF and HFrEF (31, 47, 48). SGLT2 inhibitors act by excreting sugar ingested as food in the urine and, therefore, may induce a combined risk of weight loss in patients with HF. In this sense, they may not positively impact HF outcomes in patients with low GNRI. Nevertheless, its use is strongly recommended in HF guidelines (31, 32). In the present study, the prescription rate of SGLT2 inhibitors in patients with lower GNRI was lower than in patients with higher GNRI (Supplementary Table S1). Indeed, some cases of marked weight loss, ketoacidosis, and gastrointestinal symptoms have been experienced with administration in elderly patients. Further studies are needed to evaluate the benefit of SGLT2 inhibitors and their impact on nutritional and physical indices in hospitalized elderly HF patients.

### Factors other than nutrition that affect GNRI in elderly HF patients

Albumin, used to assess GNRI, has also been reduced in HF patients due to factors other than nutrition. These factors include hemodilution due to congestion, inflammation, and reduced albumin synthesis due to a congested liver. Indeed, patients have relatively low albumin levels immediately after HF hospitalization due to the effects of intravascular volume loading, assessed low by GNRI. The intravascular capacitance decreases as HF treatment progress, and albumin is conversely thought to increase. Albumin is less likely to improve in concomitant inflammation or persistent congestive liver disease cases associated with HF (49, 50). In this regard, it should be remembered that in addition to its aspect as a nutritional indicator, the GNRI on admission also has another aspect reflecting HF severity (20, 21, 51). In contrast, GNRIs evaluated at discharge are unlikely to be influenced by the circulatory dynamics of patients with exacerbated HF. In the present study, therefore, we used the value of GNRI at discharge. Recent studies (42, 52) have shown that GNRI at discharge is a more beneficial indicator of long-term prognosis than assessment at admission, which we believe supports our assessment methodology.

We should consider that elderly HF patients who responded adequately to HF treatment are prone to reduced dietary intake due to multiple factors (53). Those factors include environmental changes due to emergency hospitalization, intestinal edema, and the influence on cognitive function (54).

## Limitation

This study has several limitations. First, as our region has an advanced aging society, it is unclear whether these results can apply to patients in other hospitals or regions. Second, this study was a single-center, retrospective study with a small sample size; there were no consecutive cases and lost follow-up. Third, this cohort was exclusively Japanese, not including other races such as African American, White, Pacific, or others. Further study is required to examine the large multicenter sample size.

## Conclusion

In hospitalized elderly HF patients, GNRI is associated with all-cause mortality or HF rehospitalization and MACCE; nutritional status assessed by GNRI was a significant predictor independent of age, cardiac parameters, and physical function. Although there are many challenges in intervention methods, improving the nutritional status of hospitalized elderly HF patients is highly desirable. It should also be recognized that HF patients with a low GNRI at discharge may have a poor prognosis at one year, even if other measures of HF severity, such as BNP, are better.

## Data Availability

The original contributions presented in the study are included in the article/**Supplementary Material**, further inquiries can be directed to the corresponding author/s.

## Supplementary material

Supplementary Figure S1 The risk score models for predicting MACCE were compared using the area under the receiver operating characteristic curves.
