# Supplementary material for: The impact of geriatric nutritional risk index on one-year outcomes in hospitalized elderly patients with heart failure
Source: Front Cardiovasc Med. 2023 May 30;10:1190548. doi: 10.3389/fcvm.2023.1190548 (PMC10267999; doi:10.3389/fcvm.2023.1190548)
Supplement: Supplementary file 1 [file Table1.pdf]

Supplement Table S1 Baseline Clinical Characteristics in GNRI cut-off value of 87.6

	GNRI < 87.6 (n = 414)	GNRI ≥ 87.6 (n = 424)	P-value
Age, years	86.0(81.0-90.0)	81.0(76.0-86.0)	<0.001
Female	218(53)	217(51)	0.679
BMI, kg/m^2	18.3(16.7-19.9)	22.2(20.4-24.6)	<0.001
Living alone	86(21)	108(26)	0.120
Returning home	252(61)	341(80)	<0.001
Nursing care insurance	221(53)	142(34)	<0.001
History of HF	178(43)	145(34)	0.011
NYHA II/III/IV (on admission)	28/105/281 (7/25/68)	52/118/254 (12/28/60)	0.010
NYHA I/II/III/IV(at discharge)	234/148/23 (56/36/6/2)	317/97/9 (75/23/2/0)	<0.001
Hospital stay, days	21(15-30.8)	19(14-25)	<0.001
**Etiology**			
IHD	87(21)	90(21)	1
VHD	138(33)	119(28)	0.100
CM	38(9)	59(14)	0.040
HHD	37(9)	48(11)	0.303
Arrhythmia	54(13)	67(16)	0.280
Other (CKD, anemia)	56(14)	51(12)	0.536
Uncertain	15(4)	9(2)	0.218
**Comorbidities**			
Hypertension	275(66)	302(71)	0.136
Diabetes mellitus	144(35)	148(35)	1
AFib	152(37)	181(43)	0.078
CKD (eGFR<60mL/min/1.73m^2)	326(79)	332(78)	0.933
COPD	81(20)	54(13)	0.008
Stroke	74(18)	76(18)	1
Orthopedic disease	128(31)	116(27)	0.287
**Medication**			
ACE-I/ARB/ARNI	238(58)	293(69)	0.001
β-blocker	263(64)	293(69)	0.093
Tolvaptan	162(39)	154(36)	0.433
Loop diuretics	257(62)	246(58)	0.232
MRAs	187(45)	215(51)	0.112
SGLT2 inhibitors	40(10)	75(18)	0.001
**Echocardiography**			
LVEF, %	52.9(28.3-64.0)	49.7(37.1-62.0)	0.098
HFrEF	108(46)	125(54)	0.139
HFmrEF	68(45)	84(55)	
HFpEF	238(53)	215(47)	
LVDd, mm	48.0(43.0-55.0)	51.0(46.0-57.0)	<0.001
LVDs, mm	34.0(28.0-44.0)	37.0(30.0-47.0)	<0.001
LAD, mm	41.0(35.0-47.0)	45.0(39.0-49.0)	<0.001

E/e’	16.2(11.8-22.2)	15.7(12.0-21.5)	0.780
TR-PG, mmHg	28.0(22.4-34.7)	26.1(20.7-34.0)	0.044
ePAP, mmHg	33.5(28.0-40.3)	32.0(26.0-40.0)	0.017
IVC, mm	14.0(11.0-17.0)	14.0(11.0-17.0)	0.987
**Laboratory data**			
BNP, pg/mL	596.0 (366.0-1090.3)	495.8 (272.6-762.8)	<0.001
Log BNP	2.78(2.56-3.04)	2.70(2.44-2.88)	<0.001
Albumin, g/dL	3.0(2.7-3.3)	3.6(3.4-3.9)	<0.001
Hemoglobin, g/dL	10.6(9.4-11.8)	11.9(10.6-13.3)	<0.001
Creatinine, mg/dl	1.1(0.83-1.55)	1.10(0.85-1.40)	0.523
eGFR, mL/min/1.73m^2	42.0(29.0-56.0)	44.0(31.0-57.0)	0.161
GNRI	80.8(75.8-84.1)	95.1(91.6-100.9)	<0.001
**Physical Function**			
SPPB, points	6(3-8.5)	8(5-11)	<0.001
QIS, Nm/kg	0.61(0.45-0.77)	0.63(0.48-0.79)	0.117
Handgrip, kg	12.3(7.8-17.1)	16.0(11.0-21.7)	<0.001
BI, points	75.0(50.0-85.0)	90.0(70.0-95.0)	<0.001
6MWT	184.0(79.0-280.5)	262.0(172.3-360.0)	<0.001

Table S1. The abbreviations are the same as listed in Table 1.
