# Supplementary figures and images for: The impact of geriatric nutritional risk index on one-year outcomes in hospitalized elderly patients with heart failure
Source: Front Cardiovasc Med. 2023 May 30;10:1190548. doi: 10.3389/fcvm.2023.1190548 (PMC10267999; doi:10.3389/fcvm.2023.1190548)

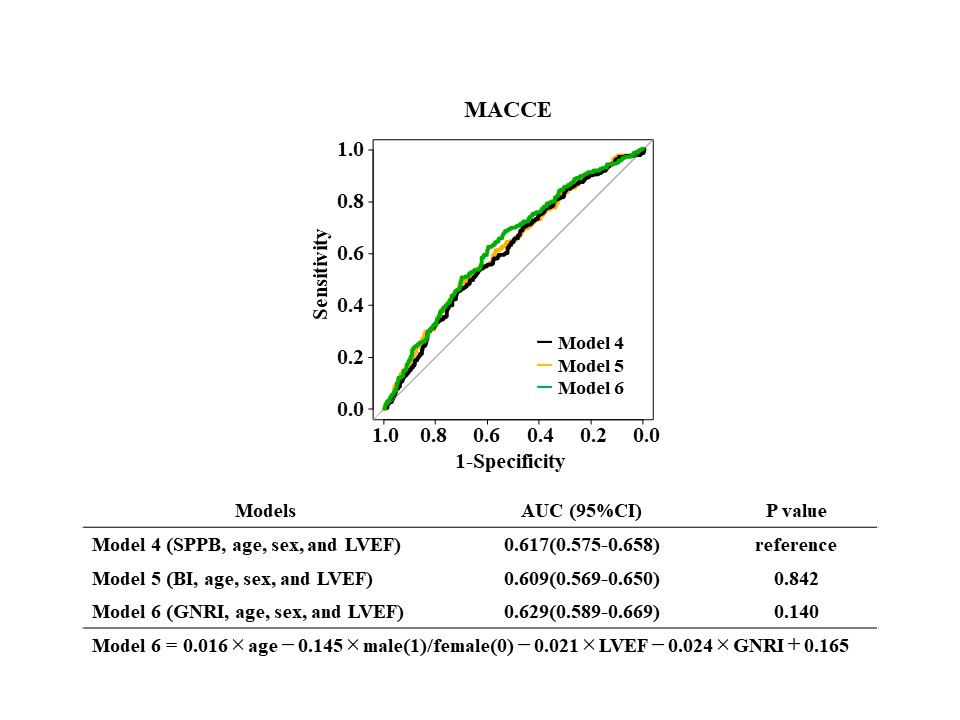

Supplement: Supplementary Figure S1 — The risk score models for predicting MACCE were compared using the area under the receiver operating characteristic curves. [file Image1.jpeg]
